# Endovascular treatment of multiple intracranial aneurysms in patients with subarachnoid hemorrhage: one or multiple sessions?

**DOI:** 10.3389/fneur.2023.1196725

**Published:** 2023-06-20

**Authors:** Guangjian Zhang, Weiwei Zhang, Hanxiao Chang, Yuqi Shen, Chencheng Ma, Lei Mao, Zheng Li, Hua Lu

**Affiliations:** ^1^Department of Neurosurgery, The First Affiliated Hospital of Nanjing Medical University, Nanjing, China; ^2^Department of Neurosurgery, Jiangsu Province Hospital, Nanjing, China; ^3^Department of Ophthalmology, Third Medical Center of Chinese PLA General Hospital, Beijing, China

**Keywords:** multiple aneurysms, subarachnoid hemorrhage, endovascular treatment, one-stage group, staged group

## Abstract

**Objective:**

This study aimed to compare the safety and efficacy of single- and multiple-stage endovascular treatment in aneurysmal subarachnoid hemorrhage (SAH) patients with multiple intracranial aneurysms.

**Methods:**

We retrospectively analyzed the clinical and imaging data of 61 patients who harbored multiple aneurysms and presented to our institution with aneurysmal subarachnoid hemorrhage. Patients were grouped according to endovascular treatment strategy: one-stage or multiple-stage.

**Result:**

The 61 study patients harbored 136 aneurysms. One aneurysm in each patient had ruptured. In the one-stage treatment group, all 66 aneurysms in 31 patients were treated in one session. The mean follow-up was 25.8 months (range, 12–47). At the last follow-up, the modified Rankin scale was ≤2 in 27 patients. In total, 10 complications occurred (cerebral vasospasm, six patients; cerebral hemorrhage, two patients; and thromboembolism, two patients). In the multiple-stage treatment group, only the ruptured aneurysm (30 in total) was treated at the time of presentation, and the remaining aneurysms (40 in total) were treated later. The mean follow-up was 26.3 months (range, 7–49). At the last follow-up, the modified Rankin scale score was ≤2 in 28 patients. In total, five complications occurred (cerebral vasospasm, four patients; and subarachnoid hemorrhage, one patient). During the follow-up period, there was one recurrence of aneurysm with subarachnoid hemorrhage in the single-stage treatment group and four recurrences in the multiple-stage treatment group.

**Conclusion:**

Both single- and multiple-stage endovascular treatment is safe and effective in aneurysmal subarachnoid hemorrhage patients who harbor multiple aneurysms. However, multiple-stage treatment is associated with a lower rate of hemorrhagic and ischemic complications.

## Introduction

Multiple intracranial aneurysm (MIA) refers to aneurysms with two or more intracranial vessels, accounting for 15–45% of all intracranial aneurysm patients (1). Aneurysmal rupture is the cause of non-traumatic subarachnoid hemorrhage (SAH) in 85% of cases, with an incidence of 9 per 1,00,000 patients/year (2, 3). SAH patients who are not treated at presentation have a 1-year mortality rate as high as 65% (4). With appropriate treatment, this rate decreases to 18% (5). Both morbidity and mortality rates have been decreasing in conjunction with advances in SAH diagnosis and treatment over time. In 2019, 400,000 people died of SAH worldwide; the highest death rate was in Asia (6).

Treatment decision-making requires a thorough evaluation of the patient's condition and comorbidities, the location of the ruptured aneurysm, and the characteristics of other aneurysms. To date, no standardized protocol for the treatment of SAH patients with multiple intracranial aneurysms has been established. The primary decision with such patients is whether to initially treat only the ruptured aneurysm and then the remaining ones at a later stage or to treat all the aneurysms in a single stage. This study aimed to compare the safety and efficacy of single- and multiple-stage endovascular treatment of patients with multiple intracranial aneurysms who present with aneurysmal rupture.

## Materials and methods

### Patients

From June 2017 to November 2022, a total of 1,191 patients with intracranial aneurysms were admitted to the Department of Neurosurgery of the First Affiliated Hospital of Nanjing Medical University, of which 256 had multiple aneurysms (95 ruptured multiple aneurysms), accounting for 21.5%. Of the 95 patients with continuous multiple intracranial aneurysmal subarachnoid hemorrhage, 34 patients were not included in this study as the remaining unruptured aneurysms remained untreated after phase I treatment of the responsible aneurysm. In total, 61 of these patients who presented with aneurysmal rupture and underwent endovascular treatment were included for analysis. In total, 40 were women and 21 were men. The mean age was 59.2 years (range, 27–80). This study was approved by the institutional ethics committee. Written informed consent was obtained from all patients.

### Diagnosis

The inclusion criteria include were (1) Patients who were diagnosed with aneurysmal subarachnoid hemorrhage on CT cranial; (2) patients who were identified with multiple intracranial aneurysms by digital subtraction angiography (DSA), computer tomography angiography (CTA), or magnetic resonance angiography (MRA); and (3) all patients who were treated with endovascular therapy to embolize the aneurysms. The exclusion criteria include were: (1) The subarachnoid hemorrhage was not caused by an aneurysm; (2) patients who suffered from other cerebrovascular disorders such as cerebrovascular malformations and Moyamoya disease; and (3) patients who chose to treat only ruptured aneurysms but left unruptured aneurysms untreated.

### Endovascular technique

In our institution, all patients undergo computed tomography and computed tomography angiography before treatment. Intravenous nimodipine (2 mg/h) is routinely administered to patients with aneurysmal rupture to reduce the incidence of delayed cerebral ischemia. Specific treatment is based on the patient's condition and comorbidities, vascular anatomy, and aneurysm characteristics. In patients who undergo stent placement, aspirin 300 mg and clopidogrel 300 mg are administered at least 30 min before the procedure. The procedure is performed under general anesthesia. Intracranial hypertension and acute changes in intracranial pressure are avoided. Blood pressure is strictly controlled during the procedure. When only the ruptured aneurysm is treated, we recommend delayed treatment of the remaining aneurysms after the patient recovers (typically at least 1 month after hospital discharge).

**Figure 1:**
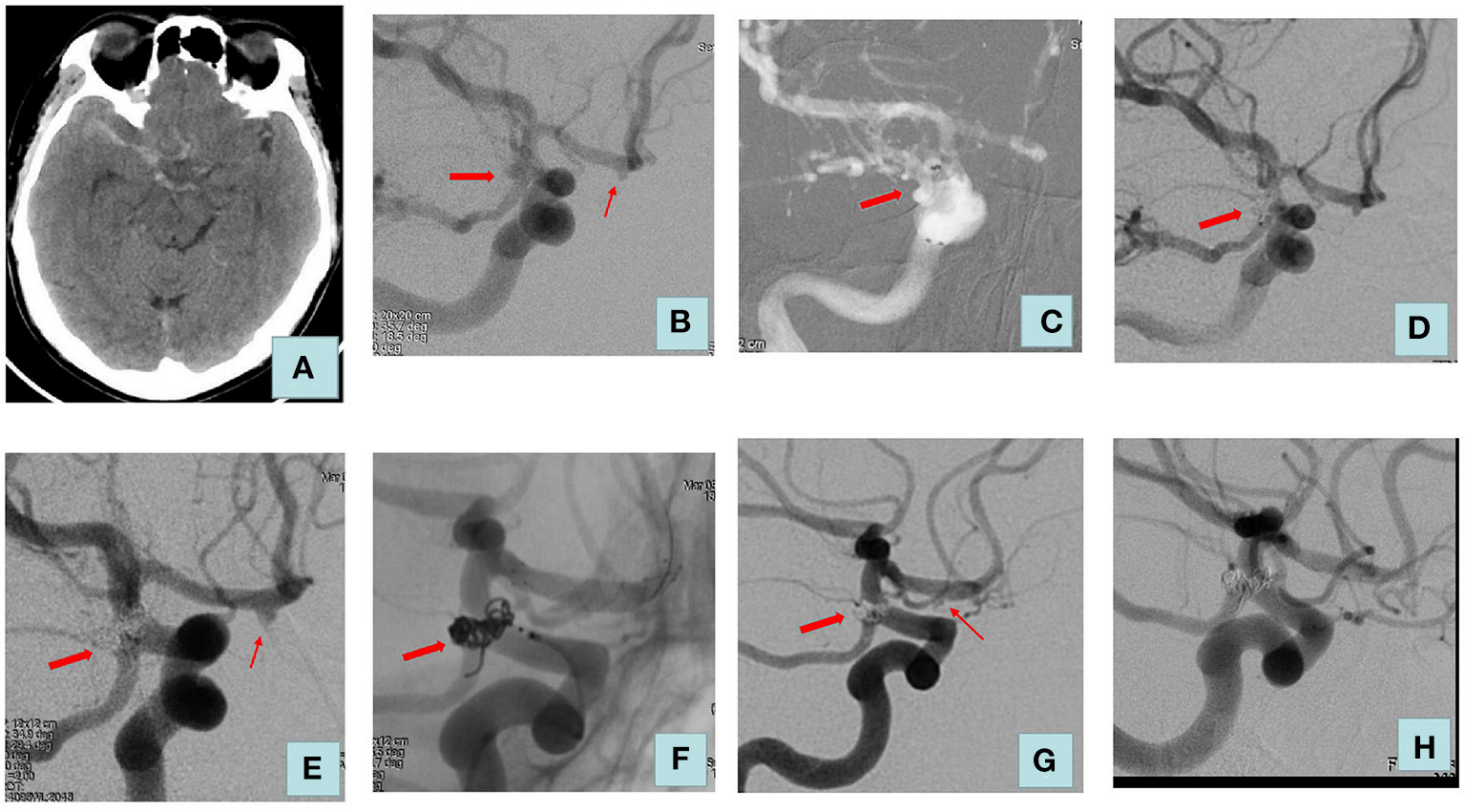
Adult patient presented with severe headache for 3 days. **(A)** Computed tomography of the head revealed a diffuse subarachnoid hemorrhage. **(B)** Cerebral angiography showed a right posterior communicating aneurysm (size: 4.2 * 3.8 mm) and an aneurysm of the right anterior cerebral artery (size: 2.2 * 1.8 mm). The posterior communicating aneurysm was considered to be a ruptured aneurysm. **(C)** Double microcatheter delivery to the aneurysm lumen. **(D)** We embolized the ruptured aneurysm using coils alone, and the Raymond–Roy classification immediately was complete embolization. **(E)** No recurrence of posterior communicating artery aneurysm at a 6-month postoperative review. **(F)** Treatment of unruptured aneurysm with one LVIS stent. **(G)** Raymond–Roy classification immediately was sac remnant (right anterior cerebral artery). **(H)** Review 2 years after surgery: posterior communicating artery aneurysm and anterior cerebral artery aneurysm were complete embolization. The thick arrow indicates a ruptured aneurysm; the thin arrow indicates an unruptured aneurysm.

**Figure 2:**
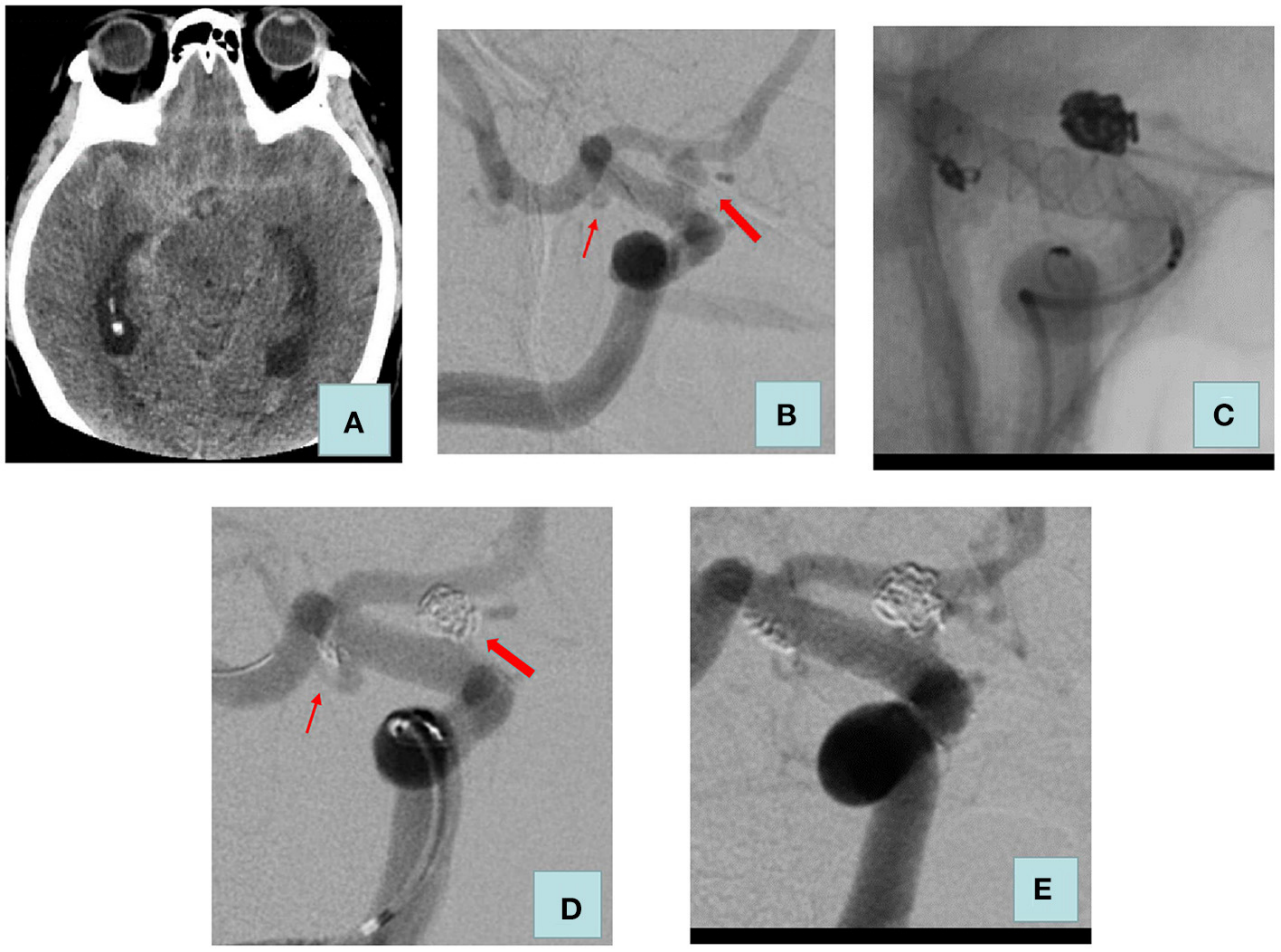
Adult patient presented with severe headache for 5 h. **(A)** Computed tomography of the head revealed a diffuse subarachnoid hemorrhage. **(B)** Cerebral angiography showed a blood blister aneurysm (size: 3.9 * 3.5 mm) and a saccular aneurysm (size: 2.1 * 1.0 mm) of the right internal carotid artery C7 segment. **(C)** We used LVIS stent-assisted coils embolizing the two aneurysms. **(D)** Raymond–Roy classification immediately was as follows: ruptured aneurysm, complete embolization, unruptured aneurysm, and sac remnant. **(E)** Review 4 months after surgery: the two aneurysms were complete embolization. The thick arrow indicates a ruptured aneurysm; the thin arrow indicates an unruptured aneurysm.

Our treatment strategy is as follows: (1) We treat all aneurysms in one stage when two aneurysms are in close proximity, and it is difficult to accurately identify the responsible aneurysm or when treating a ruptured aneurysm would have an impact on the adjacent unruptured aneurysm. (2) If the hemodynamic and morphological analysis reveals that all aneurysms are at high risk of rupture, we will opt for one-stage treatment. (3) In the remaining cases, the treatment strategy is decided by the surgeon, taking into account the patient's condition, the characteristics of the aneurysm, and the surgeon's clinical experience. We prefer a high density of embolization in patients with ruptured aneurysms to decrease the risk of recurrence. Once the aneurysm is densely occluded or further coil deployment is not possible, the procedure is terminated. After the procedure, blood pressure is maintained at basal levels. Clopidogrel 75 mg and aspirin 100 mg are administered daily for 1 month and 1 year when stents are used intraoperatively, respectively. The treatment process of a patient in the multiple-stage treatment group is detailed in Figure 1 and the treatment process of a patient in the one-stage treatment group is detailed in Figure 2.

### Clinical evaluation

Post-operative follow-up is carried out by telephone and outpatient and/or inpatient DSA at specified times, with all complications during treatment and follow-up recorded and assessed retrospectively. Outpatient follow-up is scheduled 1 month after surgery, and patients are asked to bring a recent cranial CT to assess for asymptomatic bleeding or ischemic stroke in the short term. Angiographic follow-up is performed 3–6 months after treatment and then every 1–2 years. Clinical outcome is evaluated using the modified Rankin scale (MRS) score: score 0–2 is considered favorable and score 3–6 is considered unfavorable. The angiographic outcome is evaluated using the Raymond–Roy classification: grade I, complete occlusion; grade II, neck remnant; and grade III, sac remnant.

### Statistical analysis

Statistical analyses are performed using SPSS software version 22.6 (IBM Corp., Armonk, NY, USA). Continuous data with a normal distribution are expressed as mean; continuous data with a non-normal distribution are expressed as median. Categorical data are expressed as numbers with percentages and are compared using the chi-square test. A *P*-value of <0.05 is considered to be statistically significant.

## Results

Patients' characteristics and aneurysm characteristics are summarized in Table 1. In total, the 61 study patients harbored 136 aneurysms. The number of patients with 2, 3, and 4 aneurysms was 50, 8, and 3, respectively. In total, 63 aneurysms were embolized using coils alone (39 were ruptured aneurysms). The stent utilization rate for the responsible aneurysm was higher in the one-staged treatment group (48.4%) than in the staged treatment group (23.3%). In this study, the size of ruptured aneurysms ranged from 2.1 to 14.2 mm (5.0 ± 2.4) and the size of unruptured aneurysms ranged from 1.6 to 10.2 mm (3.7 ± 1.6), with a *p*-value of < 0.05. The Raymond–Roy classification immediately after treatment was as follows: complete embolization, 97 aneurysms; neck remnant, 32 aneurysms; and sac remnant, seven aneurysms. A total of 30 patients had hypertension. Hunt-Hess grading I to III at admission was as follows: 26 cases (83.9%) in the one-staged treatment group and 24 cases (80%) in the staged treatment group. Specific information on all patients is shown in Table 1.

**Table 1:** Patient and aneurysm characteristics.

	**One-stage**	**Multiple-stage**	***P*-value**
Sex	31	30	
Male	14 (45.2%)	7 (23.3%)	0.073
Female	17 (54.8%)	23 (76.7%)	0.073
Age, years (mean)	62.2	56.0	0.025
No. of aneurysm treated	66	70	
Size of aneurysms (ruptured/unruptured)	31/35	30/40	
< 5 mm	19 (61.3%)/30 (85.7%)	16 (53.3%)/31 (77.5%)	0.364
≥5 to < 10 mm	11 (35.5%)/5 (14.3%)	13 (43.4%)/8 (20%)	0.451
≥10 mm	1 (3.2%)/0 (0%)	1 (3.3%)/1 (2.5%)	1.000
Location of aneurysms (ruptured/unruptured)	31/35	30/40	
Anterior cerebral artery	1 (3.2%)/1 (2.9%)	4 (13.3%)/3 (7.5%)	0.197
Anterior communicating artery	6 (19.4%)/2 (5.7%)	6 (20%)/3 (7.5%)	0.897
Internal carotid artery	7 (22.6%)/16 (45.7%)	6 (20%)/16 (40%)	0.672
Middle cerebral artery	2 (6.5%)/1 (2.9%)	2 (6.7%)/5 (12.5%)	0.374
Posterior communicating artery	9 (29%)/7 (20%)	9 (30%)/7 (17.5%)	0.849
Basilar artery	2 (6.5%)/3 (8.6%)	2 (6.7%)/3 (7.5%)	0.923
Posterior cerebral artery	1 (3.2%)/0 (0%)	0 (0%)/2 (5.0%)	1.000
Others	3 (9.6%)/5 (14.2%)	1 (3.3%)/1 (2.5%)	0.082
Hunt-Hess score	31	30	
I–III	26 (83.9%)	24 (80%)	0.694
IV–V	5 (16.1%)	6 (20%)	0.694
Stent assist (total aneurysms)	32 (66)	41 (70)	0.238
Ruptured aneurysms	15 (31)	7 (30)	0.042
Unruptured aneurysms	17 (35)	34 (40)	0.002
Raymond-Roy occlusion classification	66	70	
1	47 (71.2%)	50 (71.4%)	0.978
2	15 (22.7%)	17 (24.3%)	0.830
3	4 (6.1%)	3 (4.3%)	0.936

### Clinical outcomes and complications

Mean follow-up in the one- and multiple-stage treatment groups was 25.8 months (range, 12–47) and 26.3 months (range, 7–49), respectively. In total, 15 complications occurred during the peri- and postprocedural follow-up periods, 10 in the one-stage treatment group and 5 in the multiple-stage group. In the one-stage group, intraprocedural cerebral vasospasm occurred in six patients. The cause was SAH-related vascular irritation in two patients and surgical manipulation in four patients. Vasospasms improved in all six patients after nimodipine administration and retraction of the guiding catheter; none experienced a related neurological deficit. Two one-stage treatment patients developed cerebral hemorrhage: one patient was diagnosed with hypertensive cerebral hemorrhage 6 months after stent-assisted embolization. This patient improved after supportive care. The other patient experienced hemorrhage related to aneurysmal recurrence 4 months after coil embolization alone was performed for two aneurysms; at the time of recurrence, the recurrent aneurysm was embolized using stent assistance. Thromboembolic events occurred in two one-stage treatment group patients. One patient developed a right eye upper outer quadrant visual field defect that did not improve after intravenous tirofiban administration; an ophthalmologic examination suggested embolization of a central retinal artery branch. The other developed an ischemic stroke 7 months after the procedure that resolved after treatment at a local hospital. In this patient, the ruptured aneurysm was treated using coil embolization alone, while the unruptured aneurysm was treated using stent-assisted coil embolization.

In the multiple-stage treatment group, intraprocedural cerebral vasospasm occurred in four patients. The cause was SAH-related vascular irritation in three patients and surgical manipulation in one patient; none developed a related neurological deficit. SAH occurred in one multiple-stage patient. This patient, whose ruptured aneurysm was treated using stent-assisted coil embolization in the first-stage procedure, did not comply with our recommendation for timely second-stage treatment of the remaining aneurysm. After the untreated aneurysm ruptured, it was embolized using stent-assisted coil embolization. Procedural and postprocedural follow-up data are shown in Table 2.

**Table 2:** Complications and clinical and angiographic outcomes according to group.

	**One-stage group**	**Staged group**	***X*^2^-test *P***
Complications	10	5	
Vasospasm	6 (60%)	4 (80%)	0.772
Cerebral hemorrhage	2 (20%)	1 (20%)	>0.05
Thromboembolism	2 (20%)	0 (0%)	NA
Operation time (minutes)^#^	178.1	136.0	< 0.05
MRS score	31	30	
0–2	27 (87.1%)	28 (93.3%)	0.698
3–6	4 (12.9%)	2 (6.7%)	0.698
Raymond score^*^	51	53	
1	42 (82.3%)	43 (81.1%)	0.872
2	8 (15.7%)	6 (11.3%)	0.514
3	1 (2.0%)	4 (7.6%)	0.383

*,# Operation time in the staged group refers to the first treatment.

Two one-stage treatment group patients had permanent neurological deficits at the last follow-up (both with left hemiplegia), and two patients died because of severe conditions after endovascular treatment. In the multiple-stage group, two patients had permanent neurological deficits (diplegia in both lower limbs and hypertonia in all four limbs).

### Angiographic outcomes

In the one-stage treatment group, for patients mentioned above who experienced an aneurysmal recurrence that was treated using stent-assisted embolization, complete occlusion was achieved with re-treatment. In the multiple-stage group, four patients developed aneurysmal recurrence during follow-up. Two underwent stent-assisted coil embolization that achieved complete occlusion. One patient with a small sac remnant underwent observation. The remaining patient with a recurrence declined treatment and opted for clinical and imaging observation.

## Discussion

The prevalence of intracranial aneurysms in adults is ~3.2% (7). There are ~15–22% of patients with unruptured intracranial aneurysms and 20–33% of patients with aneurysmal subarachnoid hemorrhage who harbor multiple IA (3). The study by Mocco et al. (8) reported that aneurysm size and location are significant predictors of rupture. Feng et al. (9) believed that multiple aneurysms do not increase the risk of rupture of individual aneurysms, but the potential cumulative effect may improve the risk of SAH in patients with multiple intracranial aneurysms. In addition, the morbidity and mortality rates of patients with aneurysmal SAH are higher in patients with multiple aneurysms than in those with single intracranial aneurysm (SIA). In patients with multiple intracranial aneurysmal subarachnoid hemorrhages, ~2–5% of ruptured aneurysms cannot be identified, and the prognosis for these patients is usually poor.

Nehls et al. (10) noted that in patients with multiple intracranial aneurysms, the most common location of the aneurysm was the posterior communicating artery, while the anterior communicating artery aneurysm was most likely to rupture. In our study, the most common locations of aneurysms were the internal carotid artery (33.1%), followed by the posterior communicating artery (23.5%) and anterior communicating artery (12.5%), while the most common locations of ruptured aneurysms were posterior communicating artery (29.5%), followed by internal carotid artery (21.3%) and anterior communicating artery (19.7%), similar to the results reported in the literature. Backes et al. (11) reported that ruptured aneurysms were not the largest aneurysms in 29% of patients with multiple intracranial aneurysms. In our study, the size of ruptured aneurysms ranged from 2.1 to 14.2 mm (5.0 ± 2.4), and the size of unruptured aneurysms ranged from 1.6 to 10.2 mm (3.7 ± 1.6), with a *p*-value of < 0.05. There were 15 patients whose ruptured aneurysm was not their largest aneurysm (24.6%). Therefore, it seems that aneurysm size or aneurysm location alone is not an adequate predictor of aneurysm rupture. In SAH patients with multiple aneurysms, the location of the ruptured aneurysm is generally determined based on the degree and location of hemorrhage on computed tomography and the site of cerebral vasospasm on digital subtraction angiography. A clear pattern of bleeding, such as a clear lateralization, is highly accurate in providing a correct diagnosis of a ruptured intracranial aneurysm (10). However, it can be difficult to identify a ruptured intracranial aneurysm if SAH is diffusely present or if the MIA is located in the same area. Orning et al. (12) claimed that 4.3% of aneurysms were incorrectly identified in patients with multiple intracranial aneurysmal subarachnoid hemorrhage in their institution. Matouk et al. (13) suggested that high-resolution magnetic resonance angiography can be used to determine the location of the ruptured aneurysm in patients with multiple aneurysms and emphasized that the aneurysmal wall in ruptured aneurysms becomes significantly enhanced. The misidentification of ruptured aneurysms may have a significant impact on patient prognosis as patients may experience rebleeding and severe brain injury secondary to rebleeding, which are known predictors of poor outcomes (14, 15). Currently, aneurysm morphology and hemodynamic parameters are also widely used in the identification of ruptured aneurysms. Backes et al. (11) demonstrated that irregular shape and aspect ratio ≥1.3 were significantly associated with aneurysm rupture. Nehls et al. (10) found that irregularities in aneurysm morphology were more indicative of aneurysm rupture than size. Neyazi et al. (16) showed that the aspect ratio (AR) and the maximum relative resistance time (Aneurysm_RRT_max) were significantly associated with the rupture of aneurysms. Hadjiathanasiou et al. (17) found that irregularly shaped aneurysms were more likely to rupture than regularly shaped aneurysms. Tang et al. (18) developed a predictive model by studying intracranial mirror aneurysms and concluded that neck width, bleb formation, and size ratio were independent risk factors for aneurysm rupture, which yielded good results in a validation cohort. Walther et al. (19) demonstrated that the machine learning approach is better than the PHASES score for rupture prediction of UIAs, particularly for patients in geographically constrained areas.

In SAH patients with multiple aneurysms, the ruptured aneurysm should be treated aggressively and early to prevent further rupture. However, the treatment of unruptured aneurysms is controversial. It has been suggested in the literature that in patients with multiple intracranial aneurysmal subarachnoid hemorrhage, there may be an increased risk of surgical complications when treating unruptured aneurysms in the acute phase (17). In our study, 30 patients opted for staged treatment, and only one hemorrhagic stroke occurred. Li et al. (20) reported that one-stage treatment was safe regardless of whether the multiple aneurysms were in the acute phase of bleeding. In our study, compared to the staged treatment group, stroke complications were higher in the one-stage treatment group, but there was no statistical difference between the two groups (*p* > 0.05). Since the annual rupture rate of unruptured intracranial aneurysms is ~1.9% (21), some experts suggested that treating all intracranial aneurysms in one stage would not only reduce the risk of aneurysm rupture but also save patients the cost of treatment. Cheong et al. (22) analyzed over 300 cases of multiple aneurysms and found that the rate of rupture of previously unruptured aneurysms in patients with a history of SAH ranged from 0.28 to 1.63%. In addition, Hino et al. treated 76 cases of multiple aneurysms, and four of the six patients with incorrectly identified ruptured aneurysms had postoperative rebleeding (14). These results support the use of one-stage embolization for multiple aneurysms to prevent problems caused by untreated aneurysm rupture and misidentification of ruptured aneurysms. In our study, one patient was not treated promptly for an unruptured aneurysm and had a rebleeding event at follow-up. In addition, 31 patients in our center were not actively treated for the remaining aneurysms after treating only the ruptured aneurysm in phase I. They chose to follow up on unruptured aneurysms, which are still at a potential risk of rupture.

In the cases of multiple intracranial aneurysmal subarachnoid hemorrhage, both one-stage treatment and staged treatment have their advantages. For the one-stage treatment group, first, the rupture of untreated unruptured aneurysms is avoided. Second, it reduces the financial burden on the patient. One-stage procedures can provide considerable cost savings. One-stage endovascular treatment of multiple intracranial aneurysms avoids repeated general anesthesia and groin punctures compared to staged treatment. Third, it eliminates patient concern and anxiety regarding the rupture of untreated aneurysms. In our study, 10 complications occurred in the one-stage treatment group, including one SAH related to the recurrence of an embolized aneurysm using coils alone. One patient experienced thromboembolism in a central retinal artery branch. This was probably because the procedure was long and saline was not continuously infused through the introducer catheter. One patient developed a cerebral infarction 7 months after all aneurysms were treated. Multiple-stage treatment can also have advantages. First, the use of dual antiplatelet therapy in the acute post-SAH period is avoided. For the staged treatment group, we prefer to embolize the ruptured aneurysm using coiling alone in a first-stage procedure in order to avoid the use of stents (except for special types of aneurysms such as blood blister aneurysms, dissecting aneurysms, or wide neck aneurysms). Incidence rates of bleeding and ischemic complications are high in aneurysmal SAH patients who undergo stent-assisted coil embolization (23–25). Nagahama et al. (26) concluded that dual antiplatelet therapy in aneurysmal SAH patients reduces the incidence of cerebral vasospasm and delayed cerebral ischemia without increasing the risk of hemorrhagic complications. In our study, the utilization rate of stents was higher in the one-stage treatment group than in the staged treatment group for ruptured aneurysms (*P* < 0.05); however, for unruptured aneurysms, the utilization rate of stents was higher in the staged treatment group (*P* < 0.05) than in the one-stage treatment group due to our preference for stent-assisted coil embolization of aneurysms in the second stage of treatment to prevent coil dislodgement into the parent artery and to reduce the recurrence of the aneurysm. In addition, there are other treatment strategies for patients with aneurysmal SAH. Waldau et al. (27) prevented acute rebleeding by embolizing only the apex of the ruptured aneurysm in a first-stage procedure; complete embolization was performed in a second procedure. They concluded that this protocol yielded good clinical outcomes. For wide-necked aneurysms, this strategy can be considered when stent placement is contraindicated or undesirable. Second, the procedure time when treating only the ruptured aneurysm is decreased. Longer procedure time is associated with a higher incidence of ischemic and/or hemorrhagic complications. Moreover, critically ill patients often have difficulty tolerating a long procedure. In our study, five complications occurred in the multiple-stage group. One was SAH caused by the rupture of an untreated aneurysm.

Jeon et al. (28) reported on a clinical study of 167 patients (418 aneurysms in total) treated in one session. Post-operative DSA showed complete occlusion of 186 (51.8%) aneurysms. Li et al. (20) reported their experience of one-stage treatment in 33 patients (72 aneurysms). The Raymond–Roy classification immediately after treatment was as follows: complete embolization, 39 aneurysms; neck remnant, 27 aneurysms; and sac remnant, six aneurysms. In our study, the Raymond–Roy classification immediately after treatment was as follows: complete embolization, 97 aneurysms; neck remnant, 32 aneurysms; and sac remnant, seven aneurysms. In addition, given the rapid development of interventional techniques and materials, specific techniques have emerged for the treatment of specific MIAs. For adjacent ruptured multiple aneurysms, Peng et al. (29) reported that single LVIS bridging was effective and feasible in a one-stage procedure. For multiple wide carotid aneurysms located at the same site in the ipsilateral internal carotid artery, simultaneous embolization of both aneurysms with only one stent is technically feasible and economical (30).

In aneurysmal SAH patients with multiple aneurysms, patient condition, aneurysm location, size, neck width, and parent artery condition should be fully evaluated before selecting a treatment strategy. When treating only the ruptured aneurysm in a patient in whom multiple-stage treatment is planned, the rupture risk of the untreated aneurysm(s) should be fully assessed. Morphological and hemodynamic parameters can be used as indicators to assess the risk of rupture, such as maximum aneurysm diameter, aspect ratio, size ratio, wall shear stress (WSS), and oscillatory shear index (OSI), which have all been shown to have a correlation with aneurysm rupture (31–33).

There are many shortcomings in this study. (1) The sample was small and further validation with a larger sample is still needed. (2) The treatment span is long and the treatment concept may have changed due to the rapid development of endovascular intervention and material science. (3) Treatment selection bias existed. When the responsible aneurysm cannot be identified or both aneurysms are at high risk of rupture, we can only choose to treat all aneurysms in one stage.

## Conclusion

Aneurysmal SAH in patients with multiple aneurysms is a difficult clinical problem. The ruptured aneurysm should be determined and aggressively treated. Bleeding risk should be assessed in the unruptured aneurysms before proceeding with individualized treatment. Both single- and multiple-stage endovascular treatment is safe and effective in aneurysmal subarachnoid hemorrhage patients who harbor multiple aneurysms. The staged treatment group had fewer complications than the one-stage treatment group but their aneurysm recurrence rate was significantly higher. To balance the risks of stent thrombosis and antiplatelet agent-induced hemorrhage, we typically embolize the ruptured aneurysm using coiling alone in a first-stage procedure followed by treatment of any remaining aneurysms in a later stage when the patient's condition allows. However, it is necessary to enroll a larger population to define the treatment efficacy of the two groups.

## Data availability statement

The raw data supporting the conclusions of this article will be made available by the authors, without undue reservation.

## Ethics statement

Ethical review and approval was not required for the study on human participants in accordance with the local legislation and institutional requirements. Written informed consent for participation was not required for this study in accordance with the national legislation and the institutional requirements.

## Author contributions

HL: research design. GZ, WZ, HC, and YS: performed research. CM and LM: data analysis and interpretation. GZ and ZL: manuscript preparation. All authors contributed to the article and approved the submitted version.
